# Seasonal variability of water characteristics in the Challenger Deep observed by four cruises

**DOI:** 10.1038/s41598-018-30176-4

**Published:** 2018-08-07

**Authors:** Caijing Huang, Qiang Xie, Dongxiao Wang, Yeqiang Shu, Hongzhou Xu, Jingen Xiao, Tingting Zu, Tong Long, Tiecheng Zhang

**Affiliations:** 1 Institute of Deep-sea Science and Engineering, Chinese Academy of Sciences, Sanya, People’s Republic of China; 2 University of Chinese Academy of Sciences, Beijing, People’s Republic of China; 3 State Key Laboratory of Tropical Oceanography, South China Sea Institute of Oceanology, Chinese Academy of Sciences, Guangzhou, People’s Republic of China; 4 Laboratory for Regional Oceanography and Numerical Modeling Qingdao National Laboratory for Marine Science and Technology, Qingdao, People’s Republic of China; 5 School of Marine Science and Technology, Tianjin University, Tianjin, People’s Republic of China

## Abstract

Thirty conductivity-temperature-depth profiler casts in the Challenger Deep were conducted during four cruises from December 2015 to February 2017. Two cruises took place in the summer, and two in the winter. The results demonstrated that water characteristics varied seasonally. The temperature minimum values were the same between the four cruises, but its depth was noticeably shallower in the winter than that in the summer. The θ-S diagram indicated that deep water is more saline in the summer than that in winter at the same potential temperature. Mixing is more intense between 5000 and 6800 m in the summer than that in the winter. The dissipation rate and eddy diffusivity vertically averaged between 5000 and 6800 m in the summer were *ε*_*T*_ = 3.277 × 10^−8^ *m*^2^*s*^−3^ and *K*_*zT*_ = 2.58 × 10^−2^ *m*^2^*s*^−1^, respectively. The geostrophic flows below the reference level of 3000 dbar were cyclonic in the summer, travelling westwards in the northern and eastwards in the southern areas of the Challenger Deep.

## Introduction

**Figure 1:**
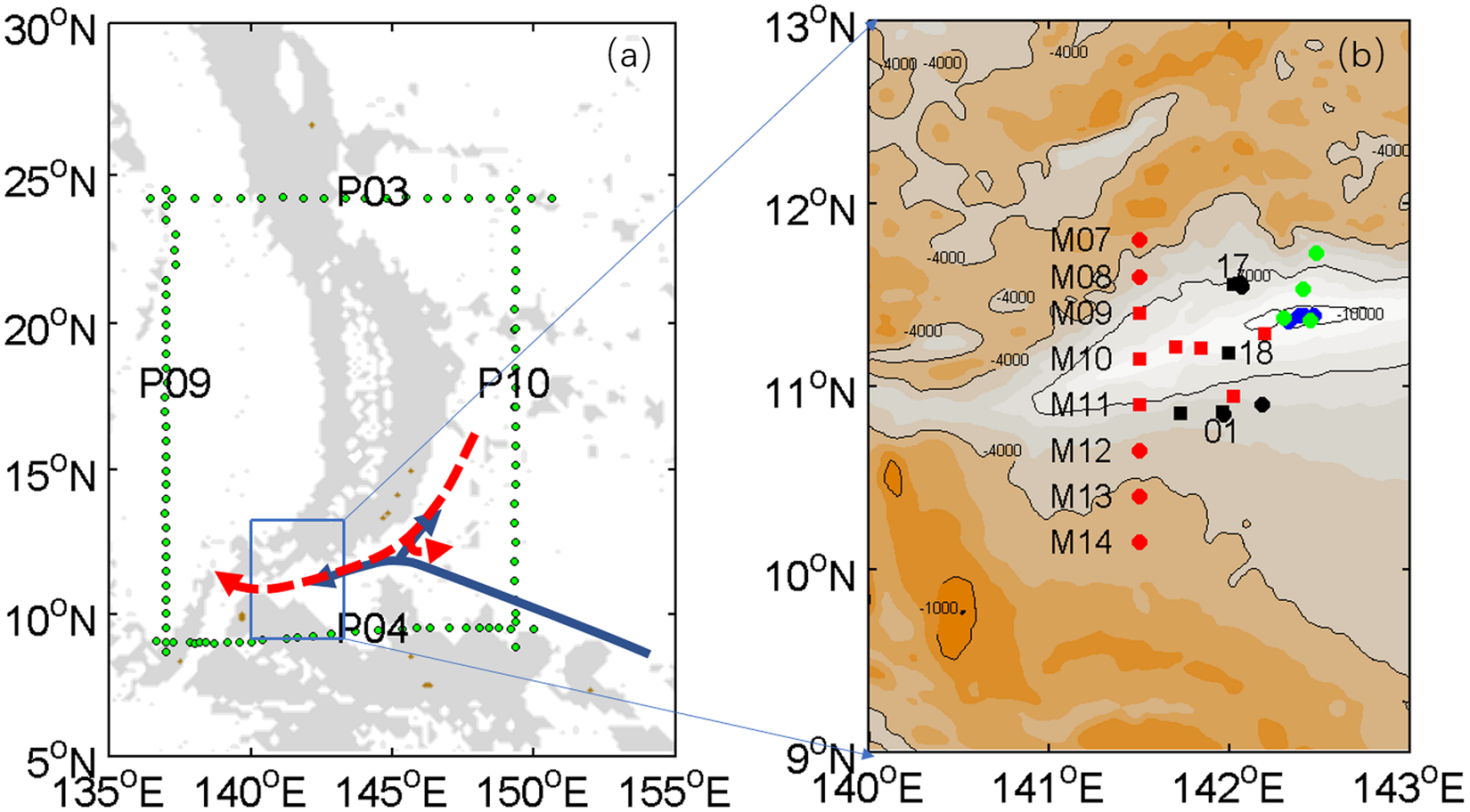
Bathymetry and locations of stations. (**a**) The transects P09, P04, P10, and P03 are CTD transects obtained from WOCE. The blue solid and red dashed arrows show the two viewpoints regarding the deep circulation near the Mariana Trench. The grey areas indicate depths between 0 and 4000 m. (**b**) The CTD stations of 2016S1, 2016S2, 2016W, and 2015W are indicated by black squares and circles, red squares and circles, blue circles, and green circles, respectively. Maps were generated using MATLAB.

The Mariana Trench, the deepest trench on the planet, is located on the western seabed of the North Pacific Ocean, east of the Mariana Islands. It is divided into two parts by a sill at 5157 m, located around 16°15′N, and the Challenger Deep is at the western edge of the southern part of the trench, extending along the east-west direction (Fig. 1). As a deep-water channel in the Philippines Sea, the Mariana Trench has a great impact on the deep and bottom circulation of the Philippine Sea.

Few previous studies focused on water mass and currents in the Challenger Deep, and they were usually based on a single voyage. Mantyla and Reid^1^ measured water characteristics in the Challenger Deep through free vehicle hydrographic casts in May 1976, and found that, below 6000 m, the trench appeared to contain a uniform water mass the same as that at the sill depth. Below the trench sill, the transect along 24°N contained a water mass that was relatively cold, saline, dense, oxygen-rich, and silica-poor^2^. Conductivity-temperature-depth profiler (CTD) casts were conducted at three stations along the north-south transect of the Challenger deep on 1 December 1992, which identified cyclonic circulation referred to 3000 dbar over the Challenger Deep^3^. With the assumed reference level of 5500 dbar, the water was flowing eastwards between the two pairs of stations in the southern area of the short meridional transect along 145°E, and westwards near the corner point^4^. However, several current meters along 142°35′E in the Mariana Trench showed a mean westwards flow^5^. CTD data collected in November 2016 revealed that the average turbulence between 6500 and 8500 m in the Challenger Deep is a magnitude of ten times higher than that between 10300 and 10850 m^6^.

It is still unknown if water characteristics in the Challenger Deep vary seasonally, because temperature, salinity, oxygen, and current are not often measured in the Mariana Trench at different times of a year. Four cruises to the Challenger Deep were conducted from December 2015 to February 2017, supported by the “Strategic Priority Research Program” of the Chinese Academy of Sciences, and aimed to measure temperature, salinity, and currents. The 2016S1 and 2016S2 cruises took place during the summer of 2016, while 2015W and 2016W cruises took place during the winters of 2015 and 2016, respectively. In this paper, the seasonal variability of water characteristics and geostrophic flow below 3000 m were obtained from the CTD data collected during these four cruises.

## Results

### Characteristics of water mass

**Figure 2:**
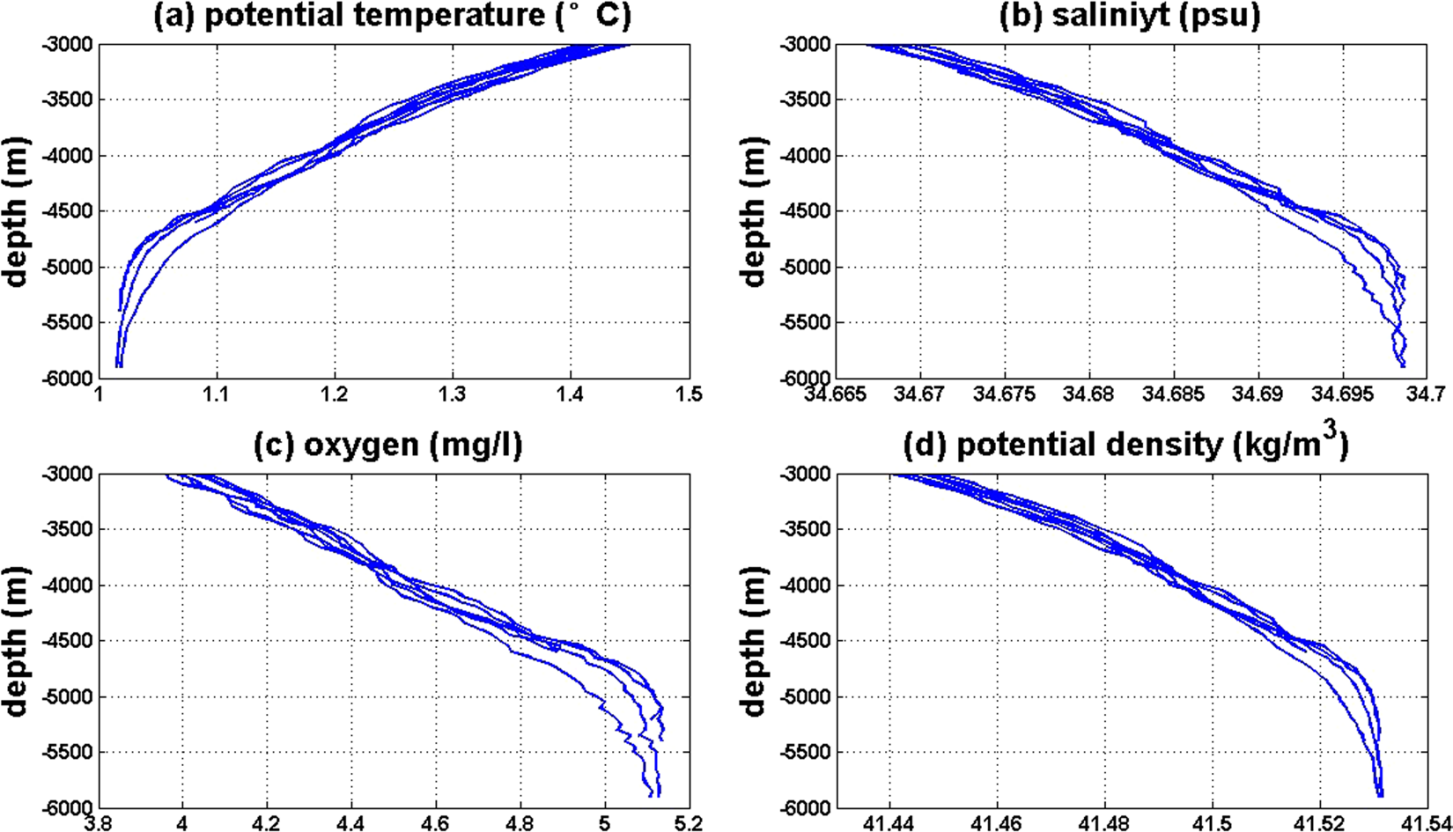
Vertical profiles of potential temperature (**a**), salinity (**b**), oxygen (**c**), and potential density (**d**) of 2016S1. Figures were plotted using MATLAB.

Figure 2 shows the vertical profiles of potential temperature, salinity, oxygen, and potential density referred to 3000 dbar from 2016S1. Below 3000 m, the potential temperature decreases to 1.02 °C at 5700 m. The salinity increases to 34.699 PSU at 5816.3 dbar. The highest oxygen content in water at the bottom layer is 5.1 mg/L(3.56 mL/L) and is smaller than 4.04 mL/L observed at 6171 dbar in 1976^1^. Below 4500 m, the rate of change is very small. The water below the trench sill is relatively dense, saline, cold, and oxygen-rich, because the bottom water in the Challenger Deep is Lower Circumpolar Water (LCPW), which is cold, saline, relatively oxygen-rich, and silica-poor. The same water mass characteristics were measured during the other three cruises.

**Figure 3:**
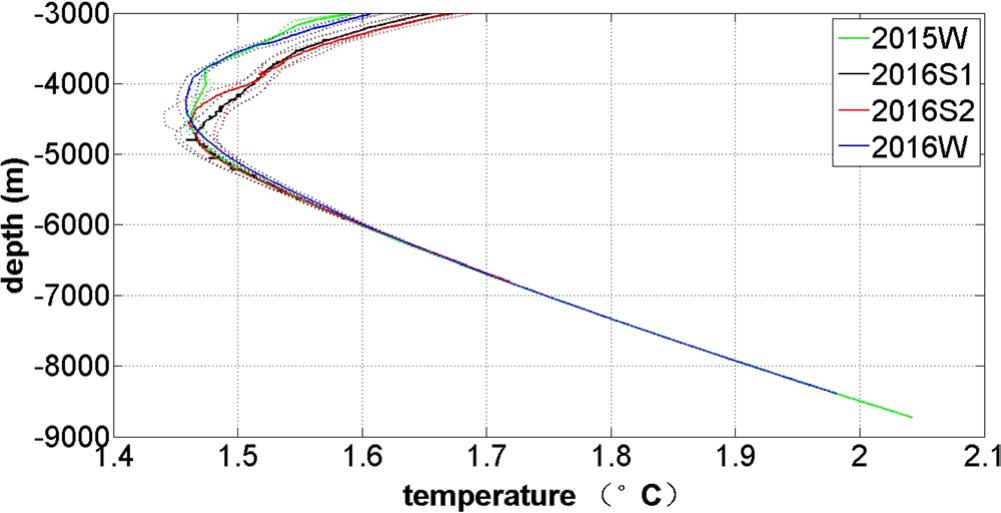
Vertical profiles of mean temperature. Vertical profiles of mean temperature below 3000 m measured at the stations during 2015W (green lines), 2016S1 (black lines), 2016S2 (red lines), and 2016W (blue lines). The dots indicate mean temperature ± standard deviation. This was generated using MATLAB.

Between the depths of 4000 and 4800 m, there is an apparent minimum temperature (Fig. 3). The temperature minimum values are almost the same between the four cruises, and is about 1.446–1.488 °C. Temperature increases to 2.042 °C at 8727.3 m in 2015W and 1.981 °C at 8389.9 m in 2016W due to adiabatic pressure^3^. The depth of the temperature minimum layer in the summer (2016S1 and 2016S2) is approximately 4700 m (4800 dbar), and 4250.5–4598.9 m in the winter (2015W and 2016W). The depth of the temperature minimum layer is shallower in the winter than that in the summer. The temperature minimum values of three stations in the winter of 1992 were 1.455–1.470 °C^3^, which is consistent with that measured during the four cruises. The minimum temperature appeared at approximately 4700 dbar in the winter of 1992, which is also shallower than that measured in the summer of 2016. In conclusion, the temperature minimum measured during the four cruises are almost same, but its depth is shallower in winter than that in summer.

**Figure 4:**
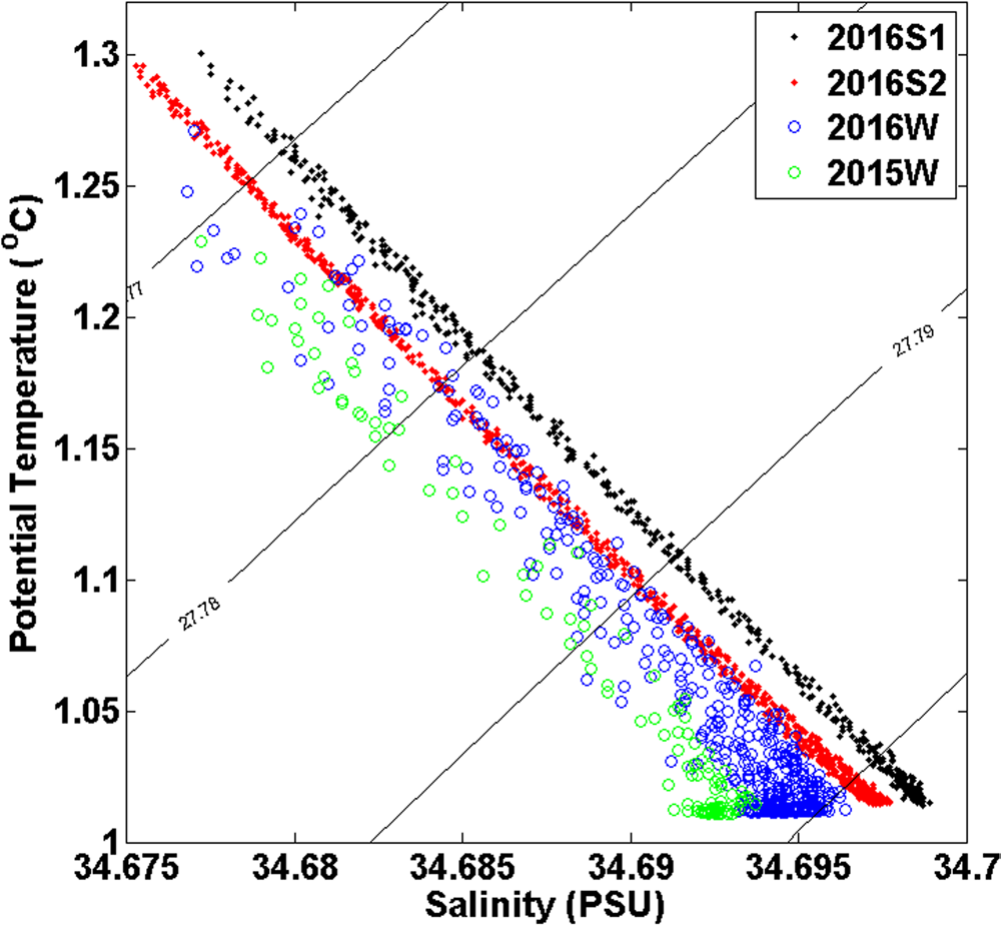
θ-S scatter diagram of the four observations. The black dots, red dots, blue circles, and green circles represent 2016S1, 2016S2, 2016W, and 2015W, respectively. Figures were plotted using MATLAB.

A θ-S diagram of the four observations is presented in Fig. 4. At the same potential temperature below 1.3 °C, the salinity measured during 2016S1 is highest, followed by 2016S2, 2016W, and 2015W. The salinity measured during 2016S1 is 0.004 PSU higher than that measured during 2015W at the same potential temperature. 2016S1 and 2016S2 were conducted during the summer of 2016. The CTD casts of 2015W and 2016W were conducted during the winters of 2015 and 2016, respectively. Therefore, deep water is more saline in the summer than that in winter at the same potential temperature.

**Figure 5:**
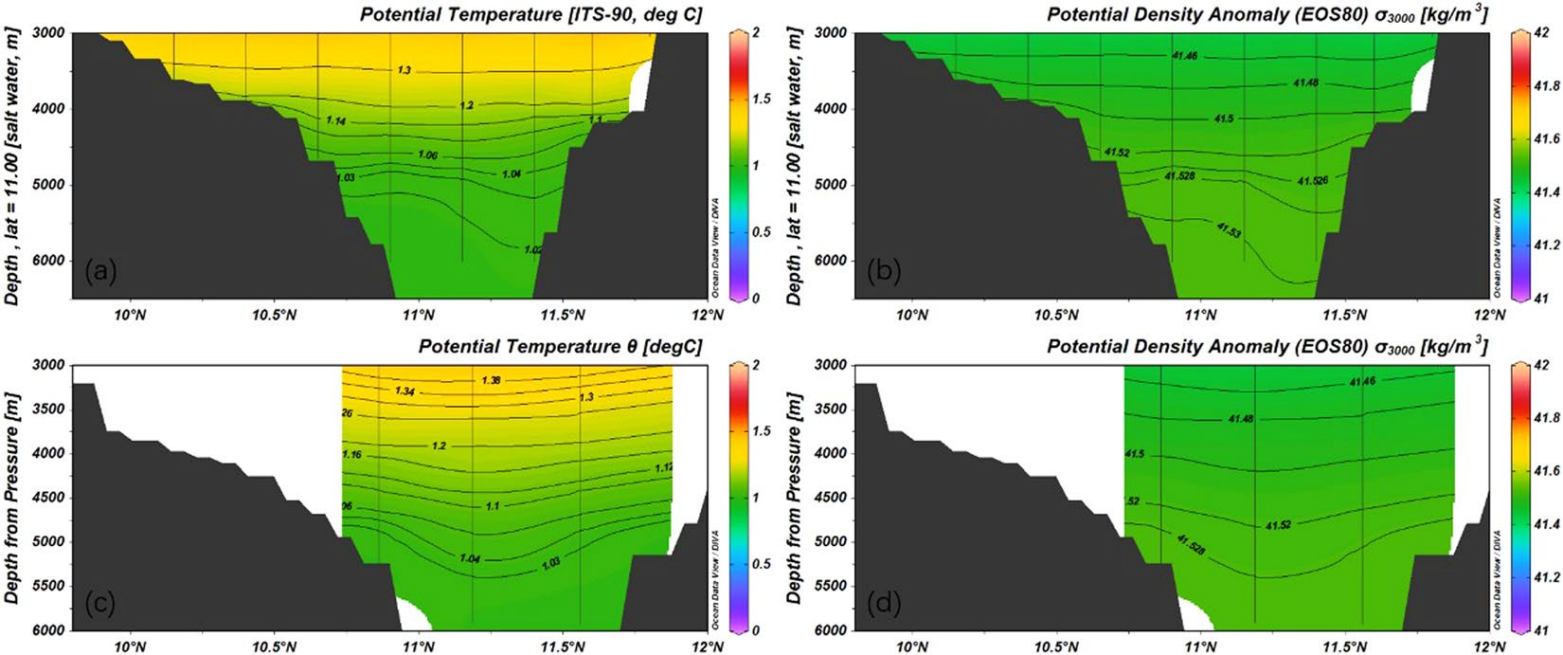
Potential temperature and potential density. (**a**) Potential temperature along the transect of 141.5°E during 2016S2, (**b**) potential density along the transect of 141.5°E during 2016S2, (**c**) potential temperature along the transect of 142°E during 2016S1, (**d**) potential density along the transect of 142°E during 2016S1. These were generated using ODV.

The contours of the potential temperature and potential density in the meridional transect of 2016S1 along 142°E (Fig. 5) are depressed over the trench. During 2016S2, the contours of potential temperature and potential density along 141.5°E are also depressed, and the depression is closer to the northern flank than that in 2016S1. In the Izu-Ogasawara Trench, the transects of potential temperatures at 34°N and 30°N are very similar^7^. However, in the Mariana Trench, although the two transects are only 54.48 km apart, the depressions of the potential temperature and potential density contours are closer to the northern flank during 2016S2 than those in 2016S1. This means that more cold water accumulates in the south along 141.5°E. Kawaba identified a maximum density of 41.517 kg/m^3^ in the Philippine Sea near the seafloor in the region closest to the deepest gap of the Yap-Mariana Junction^8^. Water that is denser than 41.52 kg/m^3^ does not reach the Philippine Sea, as the ridge prevents cold and dense water from entering. The transect along 141.5°E is closer to the ridge than that along 142°E. Therefore, more cold and dense water is accumulated in the southern area of the transect along 141.5°E.

Steep bathymetry over the Mariana Trench results in closed geostrophic regions, or f/h contours, where h is the total thickness of the water column and f is the local Coriolis parameter. The conservation of potential vorticity prevents water from flowing across geostrophic contours, and water only crosses geostrophic contours in the bottom Ekman layer^9^. This causes upwellings in the closed geostrophic regions to be smaller than those outside these regions. Therefore, the potential temperature and potential density contours are depressed over the trench. This was also found in Kawase and Straub’s experiment with the closed geostrophic contours^10^. Elevation of the fluid interface has a permanent depression over closed regions in the final state, but with a rising fluid level outsize the closed regions.

### Diapycnal mixing

**Figure 6:**
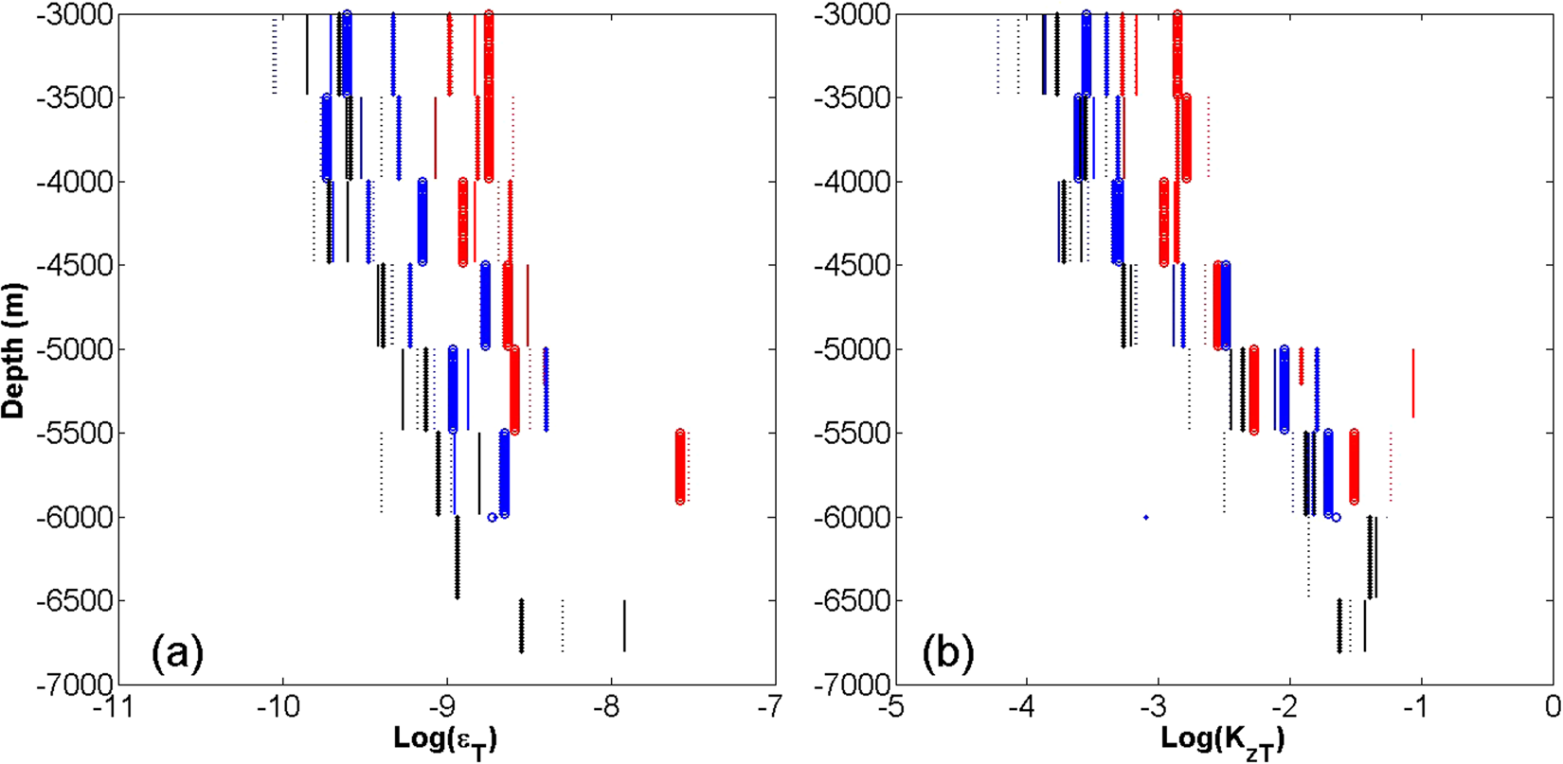
Dissipation rate and eddy diffusivity. (**a**) Logarithms of dissipation rate are computed from 11 CTD profiles deeper than 5000 m during the summer of 2016. (**b**) As (**a**), but for eddy diffusivity. The colors and shapes represent the 11 different CTD profiles. Figures were plotted using MATLAB.

The logarithm of dissipation rate and eddy diffusivity estimated from 11 sets of CTD data deeper than 5000 m (squares in the Fig. 1) during the summer of 2016 are shown in Fig. 6. Below 3000 m, the dissipation rate is almost constant. Eddy diffusivity is 10 times higher between 4500 and 6800 m than that between 3000 and 4500 m. The dissipation rate and eddy diffusivity vertically averaged between 5000 and 6800 m in the summer were ${\varepsilon }_{T}=3.277\times {10}^{-8}\,{m}^{2}{s}^{-3}$ and ${K}_{zT}=2.58\times {10}^{-2}\,{m}^{2}{s}^{-1}$, respectively. The vertical resolution of the CTD data in the winter is too low to calculate dissipation rate and eddy diffusivity. We compared the summer values with those calculated by Haren from the CTD data collected during the winter of 2016^6^. The vertical average of dissipation rate between 5000 and 7750 m derived by Haren in winter is ${\varepsilon }_{T}=2.3\pm 1.5\times {10}^{-10}\,{m}^{2}{s}^{-3}$, which is two orders of magnitude smaller than that measured between 5000 m and 6800 m in the summer of 2016. The vertical average of eddy diffusivity between 5000 and 7750 m calculated by Haren in winter is ${K}_{zT}=1.5\pm 1\times {10}^{-3}\,{m}^{2}{s}^{-1}$, which is one order of magnitude smaller than that measured between 5000 m and 6800 m in the summer of 2016. Therefore, mixing between 5000 m and 6800 m is larger in the summer than that in winter.

### Geostrophic flows

**Figure 7:**
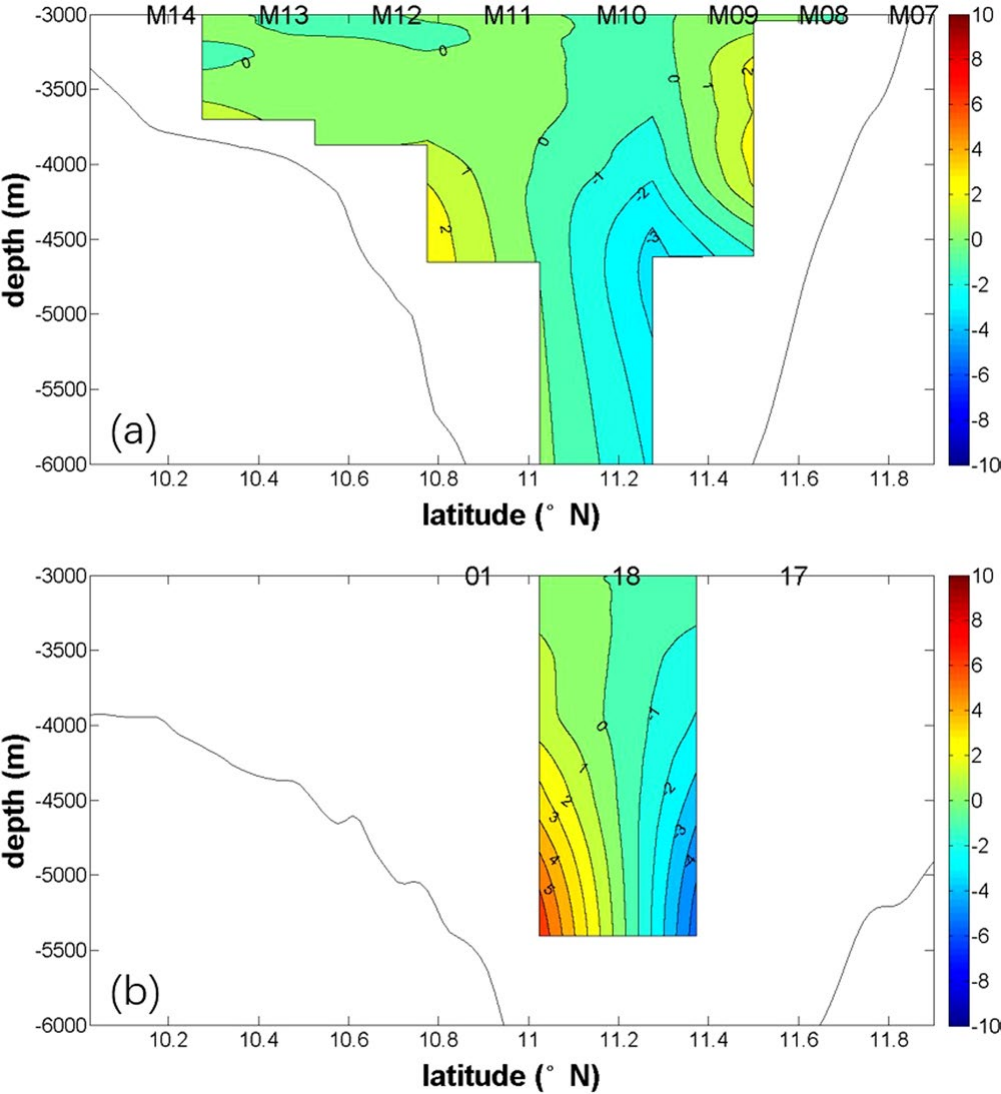
Geostrophic flow. Geostrophic velocity referred to 3000 dbar during 2016S2 (**a**) and 2016S1 (**b**). Contour intervals are 1 cm/s. Figures were generated using MATLAB.

During 2016S1 (Fig. 7b), geostrophic flows were estimated from the temperature and salinity measured at three stations. The central station is located at the Challenger Deep, at approximately 11.186°N, and the two are 36.2 km south and 41.4 km north of the central station. Figure 7b shows the geostrophic flows referred to 3000 dbar. Below 3000 m, the flow travels westwards in the north, with a maximum speed of 5.58 cm/s. In the south, the flow travels eastwards, with a maximum speed of 6.84 cm/s. The opposing geostrophic flows suggest that circulation in the Challenger Deep is cyclonic, which is same as Taria’s viewpoint that flows below 3000 dbar in the southern Mariana Trench travelled eastwards, with a maximum speed of 0.097 m/s, and westwards in the north, with a maximum speed 0.103 m/s^3^.

Geostrophic flow along the transect of 141.5°E from 9.9°N to 12°N during 2016S2 is shown in Fig. 7a. The flow is also opposite between the north and south; it travels eastwards in the south, with a maximum speed of 2.46 cm/s, and westwards in the north, with a maximum speed of 3.43 cm/s. These speeds are smaller than those measured during 2016S1. Between 3000 m and 4500 m in the area north of 11.3°N, the flow travels eastwards. In the southern area, from 10.3°N to 10.9°N, the flow at around 3200 m travels westwards.

During the summer of 2016, the circulation in the Challenger Deep is cyclonic. We cannot study the variability of the circulation, because stations do not cross the trench during 2015W and 2016W.

## Discussion

As described above, water characteristics and mixing vary seasonally. The temperature minimum values are the same between the four cruises, but its depth is noticeably shallower in the winter than that in the summer. The θ-S diagram shows that deep water is more saline at the same potential temperature in the summer than that in winter. Mixing is more intense between 5000 and 6800 m in the summer than that in the winter. The dissipation rate and eddy diffusivity vertically averaged between 5000 and 6800 m in the summer were ${\varepsilon }_{T}=3.277\times {10}^{-8}\,{m}^{2}{s}^{-3}$ and ${K}_{zT}=2.58\times {10}^{-2}\,{m}^{2}{s}^{-1}$, respectively. The geostrophic flows below the reference level of 3000 dbar were cyclonic in the summer, travelling westwards in the northern area and eastwards in the southern area of the Challenger Deep.

There are two viewpoints regarding circulation near the Mariana Trench(Fig. 1a). One is that, after moving through the Eastern Mariana Basin, LCPW branches into a western limb that moves towards the Western Basin and a northern limb towards the Northwest Pacific Basin(blue arrow in Fig. 1a)^11,12^. The other viewpoint is that, LCPW from the north is blocked by western bottom topography, which is against an inflow directly from the east. At the slope of the Mariana Trench east of Guam, a southwards western boundary current is identified, which branches into westward and an eastward return transport(red arrows in Fig. 1a)^4^. We hypothesize that the highly saline water in the Challenger Deep could have different sources between different seasons, which may result in the seasonal variability of the water mass. In the deep layer of the Challenger Deep, the highest salinity in the summer is 34.697–34.699 PSU, and highest salinity in the winter is 34.694–34.696 PSU. Four CTD transects derived from the World Ocean Circulation Experiment(WOCE) are chosen to confirm the path. The highest salinities of the bottom layer at P04, P10, P03, and P09 are 34.697, 34.700, 34.692, and 34.689 PSU, respectively. Through comparing the highest salinities in the four sections, we estimate that the water mass of the Challenger Deep in the summer originates from P10 in the east, and those in winter originate from P04 in the south. More observations are required to confirm it.

The data used in this study contains the systematic error of equipment. The accuracy of the instrument is sufficient to reveal seasonal signals, but it is not enough to quantify the systematic error due to different equipments being used in the different cruises. This requires more observation data to verify.

## Data and Methods

The 30 CTD casts deeper than 3000 m were conducted during four cruises by the Institute of Deep-sea Science and Engineering, Chinese Academy of Sciences. The four cruises are labelled as 2015W, 2016S1, 2016S, and 2016W, respectively. The stations of the four cruises are shown in Fig. 1. The time and instruments used for the four cruises are shown in Table 1. The accuracy of the SBE 16plus recorder is 0.005 °C for temperature and 0.0005 S/m for conductivity. The accuracy of the SBE 911plus system is 0.001 °C for temperature and 0.0003 S/m for conductivity. The method of data processing and correction is provided in the Supplementary information.

**Table 1:** The time and instruments of the four cruises.

Cruise	Period	Instrument	Number of CTD casts
2015W	6 December 2015 to 13 January 2016	SBE 16plus	4
2016S1	9 June to 28 June 2016	SBE 911plus with oxygen sensor	8
2016S2	29 June to 1 August 2016	SBE 911plus	13
2016W	24 February to 26 February 2017	SBE 16plus	5

Four sections of the World Ocean Circulation Experiment (WOCE) were chosen to confirm the path of high-salinity water. The four sections are as follows: P4 along 10°N from 137°E to 150°E, P10 along 149°E from 10°N to 24°N, P3 along 24°N from 137°E to 150°E, and P9 along 137°E from 10°N to 24°N. P04 was studied from February 6 to March 9 1989, P10 was studied from May 25 to July 2 2005, P03 was studied from May 4 to June 3 1985, and P09 was studied from July 6 to August 22 2010.

The geostrophic velocity was calculated using the thermal wind relation^13^:

1 $${\rm{u}}={u}_{0}+\frac{g}{f{\rho }_{0}}{\int }_{{z}_{0}}^{z}\frac{\partial \rho }{\partial y}dz^{\prime} $$

2 $${\rm{v}}={v}_{0}-\frac{g}{f{\rho }_{0}}{\int }_{{z}_{0}}^{z}\frac{\partial \rho }{\partial x}dz^{\prime} $$

where (u, v) and (${u}_{0}$,$\,{v}_{0}$) are the geostrophic velocities at depth z and at the reference level ${z}_{0}$, respectively; ${\rm{\rho }}$ is the potential density; ${\rho }_{0}$ is the characteristic potential density; and g is gravitational acceleration. The reference level is 3000 dbar^3^.

Turbulence dissipation rate ${\varepsilon }_{T}$ and vertical eddy diffusivity ${K}_{zT}$ were estimated from CTD data following the method proposed by Thorpe^14^.

3 $${\varepsilon }_{T}={c}_{1}^{2}{d}^{2}{N}^{3}$$

4 $${K}_{zT}={m}_{1}{c}_{1}^{2}{d}^{2}N$$

where ${c}_{1}=0.8$ represents the Ozmidov/overturn scale factor^15^ and ${m}_{1}$ = 0.2 is the mixing efficiency^16^. d is the displacement between the measured and reordered profiles, and N is the buoyancy frequency computed from the reordered CTD profiles.

### Data availability

The 30 CTD datasets analysed during the current study are available from the corresponding author on reasonable request.

## Electronic supplementary material

Supplementary information
