# Supplementary material for: Seasonal variability of water characteristics in the Challenger Deep observed by four cruises
Source: Sci Rep. 2018 Aug 7;8:11791. doi: 10.1038/s41598-018-30176-4 (PMC6081482; doi:10.1038/s41598-018-30176-4)
Supplement: Supplementary file 1 — Supplementary information [file 41598_2018_30176_MOESM1_ESM.pdf]

Supplementary information

Seasonal variability of water characteristics in the Challenger Deep observed by four cruises

Caijing Huang, Qiang Xie*, Dongxiao Wang*, Yeqiang Shu, Hongzhou Xu, Jingen Xiao, Tingting Zu, Tong Long & Tiecheng Zhang

The CTD raw data were processed using the standard procedures of SBE software, including wild edit, filter, cell thermal mass, and loop edit. After that, the same rules were applied to ensure that the CTD data were of good quality. First, temperatures higher than 35 °C or smaller than 0 °C were excluded. Second, salinity data higher than 36 PSU or smaller than 32 PSU were excluded. Third, data deviating from the mean by more than twice the standard deviation were excluded.

Apparently problematic data were collected during 2016S2. At station M09, the salinity suddenly decreases by approximately 0.004 PSU from 5136 m to 5138 m (figure a1). This is because the acoustic recorded exploded. In comparison with the stations (M06, M07, M08, M11, M13, M14, and M15) occupied before M09, the salinity measured at stations (M10 and M12) occupied after M09 is 0.004 PSU smaller. When calculating the potential density, the salinity of stations M10 and M12 was replaced by that of station M11. The salinity of station M09 below 5138 m is corrected by the addition of 0.004 PSU.

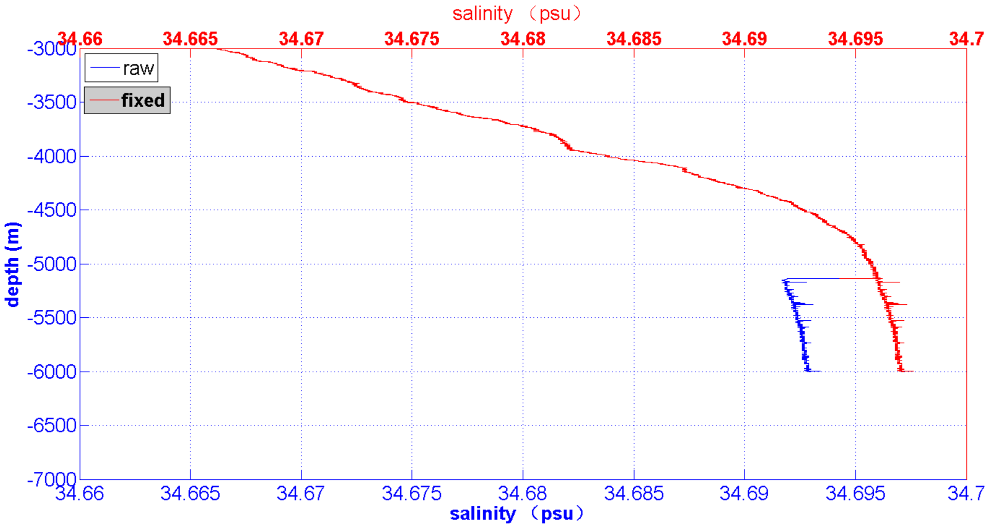

Figure a1. The blue line is the raw salinity data from station M09 during 2016S2, and the red line is the corrected salinity data.
